# Closing of the Medical Schools

**Published:** 1892-03

**Authors:** 


### THE CLOSING OF THE MEDICAL SCHOOLS.

This issue of the JOURNAL is held back a few days on account of the Commencements of the Medical Schools in this city. The session just finished has been the most successful one in the history of the two Atlanta schools, whether measured by the student patronage or the work accomplished by both the teachers and the taught. This is particularly gratifying and encouraging, from the fact that the session just past was begun with increased rates of tuition, which, it was feared, might diminish the student attendance. The amount of clinical material for lecture and operation purposes has been greater than ever before.

The commencement exercises of the Atlanta Medical College were held at DeGive's Opera House on the evening of March 2d, at 8 P. M. The exercises were opened with prayer by Rev. J. W. Pogue. The Proctor, Dr. W. S. Kendrick, then read the annual report of the faculty. He reported a number of improvements made in the college building during the past year, one of the most notable being the construction of one of the best operating and lecture amphitheatres in the country. The recently added School of Pharmacy had met with very gratifying success. The college still claimed to hold the position of first in the South. Despite an increase of twenty-five per cent. in the fees, the attendance at the last session was larger than ever. One hundred and eighty-one students had been in attendance, representing Georgia, Alabama, South Carolina, North Carolina, Texas, Florida, Louisiana, New York, Tennessee, Mississippi and Ohio. There was one graduate in pharmacy. Hon. N. J. Hammond conferred the degrees, sixty-four students receiving diplomas. The address of the occasion was delivered by Judge Howard Van Epps. Dr. J. A. S. Chambers delivered the valedictory address. Hon. E. W. Martin delivered the prizes. First honor—Dr. V. H. Taliaferro, of Atlanta. Second honor—Dr. H. W. Terrell, Greenville, Ga. Third honor—Dr. J. H. Latimer, Hazlehurst, Ga. Honorable mention—Drs. A. F. Harrington, T. V. Hubbard, J. F. Yarbrough, W. A. Sheldon, S. W. Hart.

The exercises of the Southern Medical College Commencement were held in the opera house, March 3d, and thirty-four young men were graduated. The valedictory address was delivered by Dr. George Brown, of Georgia. Dr. Jos. J. Waller, of Tennessee, was the recipient of the first prize. The second was awarded to Dr. William J. Cox, of Georgia. Dr. Thos. B. Bonner, of Georgia, received the third prize. The report of the Dean, Dr. William Perrin Nicolson, was gratifying and encouraging. One hundred students had been enrolled during the session. The increased tuition rates which were established before the beginning of the term had had the effect only of improving the *personnel* of the students in attendance. Less desirable students have sought their medical education in colleges where it is dispensed more cheaply. The faculty of the college is actively engaged on the new building, and will probably occupy it at the opening of the fall term.

We rejoice in the prosperity of these two schools and hope that the results of the past session will be progressively continued.

